# Profile of hematological abnormalities of Indian HIV infected individuals

**DOI:** 10.1186/1471-2326-9-5

**Published:** 2009-08-13

**Authors:** Byomakesh Dikshit, Ajay Wanchu, Ravinder Kaur Sachdeva, Aman Sharma, Reena Das

**Affiliations:** 1 Department of Internal Medicine, Postgraduate Institute of Medical Education & Research, Chandigarh, India; 2 Departments of Hematology, Postgraduate Institute of Medical Education & Research, Chandigarh, India

## Abstract

**Background:**

Hematological abnormalities are a common complication of HIV infection. These abnormalities increase as the disease advances. Bone marrow abnormalities occur in all stages of HIV infection.

**Methods:**

Two hundred HIV infected individual were screened for hematological abnormalities from March 2007–March 2008. Absolute CD4 cell count analysis was carried out by flowcytometry. Depending on the results of the primary screening further investigations were performed, like iron studies, hemolytic work up, PNH work up and bone marrow evaluation. Other investigations included coagulation profile, urine analysis, blood culture (bacterial, fungal, mycobacterial), serology for Epstein Barr virus (EBV), Cytomegalovirus (CMV), Hepatitis B and C, and Parvo B19 infection.

**Results:**

The most common hematological abnormality was anemia, seen in 65.5% (131/200) patients. Iron deficiency anemia was seen in 49.2% (/200) cases while anemia of chronic disease occurred in 50.7% (/200) cases. Bone marrow evaluation was carried out in 14 patients out of which staging marrow was performed in 2 cases of non-Hodgkin's lymphoma (NHL) and did not show any bone marrow infiltration. In remaining12 cases bone marrow was done for evaluation of pancytopenia. Among patients with pancytopenia 50% (6/12) showed granulomas (4 were positive for AFB, 2 were positive for fungal cryptococci), 25% (3/12) showed hemophagocytosis. There was a strong negative correlation between anemia and CD4 counts in this study. Thrombocytopenia was seen in 7% (14/200) cases and had no significant correlation with CD4 counts. No patient had absolute neutrophil count (ANC) < 800 cells/μL. No case of coagulation abnormalities was found.

**Conclusion:**

Anemia in HIV patients can be a good clinical indicator to predict and access the underlying immune status. Patients should be investigated for hematological manifestations and appropriate steps should be taken to identify and treat the reversible factors.

## Background

Hematological abnormalities are among the most common complications of HIV. These involve all lineages of blood cells [1]. HIV associated hematological abnormalities seem to be dependent on the level of virus replication, as these abnormalities are severe in late-stage AIDS patients with high viremia. The mechanism underlying these abnormalities is still obscure. A specific diagnosis of the cause and mechanism must be sought because specific treatment may be needed for its correction [1].

Anemia is the most common hematological abnormality in HIV seropositive patients and its incidence is strongly associated with the progression of the disease. Neutropenia is common in the advanced stages of AIDS and often caused or exacerbated by concomitant myelosuppressive drugs. Adverse drug reactions and their complications can cause neutropenia in patients with HIV/AIDS. Thrombocytopenia is correlated with low CD4 cell count and older age [2-4]. Bone marrow abnormalities are found in all stages of HIV disease, increasing in frequency as the disease progresses. A number of characteristic but nonspecific, morphologic abnormalities of the bone marrow of AIDS patients have been reported [5]. Bone marrow examination may be useful for the definitive assessment of iron stores which can assist in the differentiation of iron-deficiency anemia from anemia of chronic disease. There is paucity of data from India on the hematological manifestations of HIV which prompted us to conduct this study. We systematically assessed 200 HIV infected individuals attending immunodeficiency clinic, PGIMER, to look for various hematological manifestations.

## Methods

Two hundred HIV seropositive consecutive individuals were enrolled from the Immunodeficiency Clinic of Internal Medicine Department of Post Graduate Institute of Medical Education & Research (PGIMER), at Chandigarh between March 2007 to March 2008. Written informed consent was obtained from all. Patients were excluded if they refused to become part of study, were of less than 16 years of age or were pregnant.

Detailed history, general and systemic examination was carried out with emphasis on signs suggesting hematological system involvement like pallor, jaundice, edema, lymphadenopathy, koilonychias, angular cheilitis, glossitis, ecchymosis/purpura, sternal tenderness, hepatosplenomegaly and peripheral neuritis.

The investigations included complete hemogram with peripheral blood picture, complete biochemistry. Depending on the results of the primary screening further investigations were carried out – like iron studies, hemolytic work up, Paroxysmal nocturnal hemoglobinuria (PNH) work up and bone marrow study. Other investigations included coagulation profile, urine analysis, blood culture (bacterial, fungal, mycobacterial), serology for EBV, CMV, Parvo B19) and viral markers (HB_S_Ag & Anti HCV). Absolute CD4 cell count analysis was carried out by flowcytometry. All 200 patients had been divided into two groups, those having CD4 counts less than 200 cells/μL (Group 1) and those with CD4 counts more than 200 cells/μL (Group 2).

Anemia was defined as hemoglobin <13 g/dl (Men) and <12 g/dl (women). Leucopenia was defined as total WBC count less than 4000 cells/μl. Neutropenia was defined as absolute neutrophil count <1000 cells/μl. Lymphopenia was considered when absolute lymphocyte count <800 cells/μl. Thrombocytopenia was defined as total platelet count < 150 × 10^3^/μl [6-8].

### Bone marrow study

Bone marrow study was carried out in case of non-responding anemia, thrombocytopenia, pancytopenia and suspected hematological malignancy. As per indications of bone marrow study, posterior superior iliac spine was the site for bone marrow aspiration and biopsy. Bone marrow smears were stained with May Grunwald's-Geimsa stain and examined by a hematopathologist. Bone marrow samples were carefully evaluated for cellularity, differential counts, dysplastic changes, fibrosis, granulomas, organisms and iron stores. Special stains were done whenever indicated which included cytochemical stains like myeloperoxidase (MPO), periodic acid schiff's (PAS), perl's and leucocyte alkaline phospatase (LAP) stain. Reticulin stain on trephine biopsy was also performed. The bone marrow aspirates were sent for bacterial, fungal and AFB cultures.

### Statistical analysis

Descriptive statistics was expressed as mean ± SD (Range). Comparisons between data were done by Student's t test and chi-square test. A p value < 0.05 was taken as statistically significant.

The study was carried out after obtaining permission from the Institute's Ethics Committee.

## Results

The mean age of 200 HIV infected individual was 36.6 ± 8.7 yrs. One hundred eighty seven (93.5%) patients were infected due to unprotected heterosexual exposure. About two third of individuals, 67.5% (n = 135) were males and 32.5% (n = 65) were females. One hundred sixty five individuals were symptomatic and 35 were asymptomatic. Symptomatic patients presented with fever (54.5%, n = 90), generalized weakness (18.1%, n = 30), diarrhea (10.3%, n = 17), loss of weight/appetite (10.3%, n = 17) and others (6.6%, n = 11) with non specific symptoms. Out of 165 symptomatic patients, 137 patients had more than one symptom. Table 1 summarizes the results of hematological parameters evaluated in 200 HIV infected individuals. Table 2 shows the comparison between parameters and CD4 counts in asymptomatic and symptomatic groups. Mean CD4 cell count was 202.4 ± 182 cells/μL (range 8–1078 cells/μL). Sixty six individuals (33%) were receiving ART while 134 (67%) were not receiving ART.

**Table 1:** Comparison of Baseline parameters in HIV infected males & females

**PARAMETER**	**MALE (MEAN ± S.D.)** **N = 135**	**FEMALE (MEAN ± S.D.)** **N = 65**	**TOTAL** **(MEAN ± S.D.)** **N = 200**	**P value**	Institute reference values*
AGE(yrs)	37.4 ± 8.25(18–60)	35.11 ± 9.66(18–60)	36.66 ± 8.75(18–60)	0.083	

Hb (gm/dl)	10.58 ± 2.88(5.1–15.8)	9.8 ± 2.22(6.2 – 14.2)	10.3 ± 2.7(5.1–15.8)	0.081	12–18

PCV%	37.8 ± 7.4(20 – 68.4)	35.9 ± 6.7(23.6–46.6)	37.2 ± 7.2(20–68.4)	0.083	36–54

MCV(fl)	81.3 ± 8.3(62.5–103.3)	77.7 ± 7.6(62.4–94)	80.1 ± 8.3(62.4–03.3)	0.003	80–96

MCH(pg/cell)	26.3 ± 4.3(18.2–36.1)	24.2 ± 4.1(18.4 – 32.8)	25.6 ± 4.4(18.2–36.1)	0.001	27–32

MCHC%	32.2 ± 2.7(26.9–38.4)	31 ± 2.7(26.5 – 37.0)	31.8 ± 2.7(26.5–38.4)	0.005	30–36

TLC(cells/μl)	6985 ± 2740(2200–16,000)	6715 ± 2481(2800–16,000)	6898 ± 2655(2200–16,000)	0.501	4000–11000

ANC(cells/μl)	4743 ± 2090(1012–2848)	4743 ± 2090(2240–11700)	4696 ± 2052(1012–12848)	0.637	

ALC(cells/μl)	1778 ± 740(460–4200)	1722 ± 676(392–3680)	1759 ± 719(392–4200)	0.606	

PLATELETS(×10^3 ^μl)	235.1 ± 105.8(16–685)	249.5 ± 913.5(13.6–495)	239.8 ± 101.3(13.6–685)	0.348	150–400

RETIC%	1.1 ± 0.8(0.4–5.5)	1.2 ± 0.9(0.4 – 4.2)	1.17 ± 0.87(0.5–5.5)	0.318	0.2–2.0

S. IRON(μg/dl)	76.7 ± 54.2(14 – 266)	56.1 ± 46.7(14–223)	69.5 ± 52.5(14–266)	0.035	

TIBC(μg/dl)	396.3 ± 117.4(155 – 838)	404.6 ± 100.2(249–670)	399.2 ± 111.3(155–838)	0.692	

S. FERRITIN(μg/dl)	522.7 ± 464.5(10–1500)	232.6 ± 328.8(5–1420)	421 ± 443(5–1500)	0.001	

SATURATION%	20.3 ± 14.2(3.1–79)	14.5 ± 12.7(2.8–72.6)	18.3 ± 13.9	0.001	

CD4 COUNTS(cells/μL)	187.2 ± 151.1(8–785)	233.8 ± 231.8(19–1078	202.4 ± 182 (8–1078)	0.090	

*Institutional reference values have been provided by a personal Communication with Dr Man Updesh Singh Sachdeva, Department of Hematology, PGIMER, Chandigarh, India.

**Table 2:** Comparison between parameters and CD4 counts between asymptomatic and symptomatic groups

**PARAMETERS**	**Asymptomatic** **(n = 35)**	**Symptomatic** **(n = 165)**	**p value**	**TOTAL**
AGE (yrs)	36.8 ± 9.6 (22–60)	36.6 ± 8.5 (18–60)	0.864	36.66 ± 8.75 (18–60)

BMI	20.4 ± 1.8 (16–27)	19.6 ± 1.7 (15–27)	0.001	20.68 ± 1.8 (15–27)

Hb (gm/dl)	13.3 ± 1.5 (9.4–15.8)	9.7 ± 2.4 (5.1–14.5)	< 0.001	10.3 ± 2.7 (5.1–15.8)

PCV %	43.4 ± 2.5 (38.3–48.6)	35.9 ± 7.2 (20–68.4)	< 0.001	37.2 ± 7.2 (20–68.4)

MCV (fl)	85.7 ± 4.5 (67.3–95)	79 ± 8.4 (62.4–103.3)	< 0.001	80.1 ± 8.3 (62.4–103.3)

MCH (pg/cell)	28.4 ± 2.2 (18.8–32.6)	25.1 ± 4.5 (18.2–36.1)	< 0.001	25.6 ± 4.4 (18.2–36.1)

MCHC %	33.1 ± 0.01 (27.9–36)	31.5 ± 0.2 (26.5–38.4)	0.002	31.8 ± 2.7 (26.5–38.4)

TLC (cells/μl)	6560 ± 1413 (4300–10,700)	6969 ± 2848 (2200–16,000)	0.409	6898 ± 2655 (2200–6,000)

ANC (cells/μl)	4312 ± 962 (2580–5166)	4777 ± 2209 (1012–12848)	0.224	4696 ± 2052 (1012–12848)

ALC (cells/μl)	1800 ± 484 (1024–2940)	1751 ± 760 (392–4200)	0.713	1759 ± 719 (392–4200)

PLATELETS (×10^3^/μl)	248.6 ± 845.7 (150–417)	237.9 ± 104.6 (13.6–685)	0.574	239.8 ± 101.3 (13.6–685)

RETIC %	1 ± 0.4 (0.5–3.4)	1.1 ± 0.9 (0.4–5.5)	0.415	1.17 ± 0.87 (0.5–5.5)

S. IRON (μg/dl)	64.2 ± 36.0 (33–110)	69.7 ± 53.0 (14–266)	0.839	69.5 ± 52.5 (14–266)

TIBC (μg/dl)	479.2 ± 217.7 (292–752)	396.6 ± 106.8 (155–838)	0.145	399.2 ± 111.3 (155–838)

S. FERRITIN (μg/dl)	553.2 ± 545.3 (5–1280)	417 ± 441.3 (5–1500)	0.547	421 ± 443 (5–1500)

CD4 COUNTS (cells/μl)	386.6 ± 214.3 (65–1078)	163.3 ± 148 (8–967)	< 0.001	202.4 ± 182 (8–1078)

### Laboratory analysis

Anemia was found in 131 (65.5%) individuals and iron studies were done in 126. Of these 131 individuals, 92.3% (n = 121) were in Group 1, while 7.7% (n = 10) were in Group 2. Hemoglobin (Hb), Packed cell volume (PCV), Mean Cell Volume (MCV), Mean corpuscular hemoglobin (MCH), Mean corpuscular hemoglobin concentration (MCHC) (*p *< 0.001) and serum ferritin (*p *< 0.05) showed statistically significant correlation with CD4 counts and other parameters did not have significant correlation with CD4 counts (Table 3).

**Table 3:** Correlation between parameters and CD4 counts in 200 patients

**PARAMETER**	**MEAN ± S.D**	**PEARSON CORRELATION**	**P VALUE**
AGE (yrs)	36.66 ± 8.75 (18–60)	0.069	0.330

Hb (gm/dl)	10.3 ± 2.7 (5.1–15.8)	0.660**	< 0.001

PCV %	37.2 ± 7.2 (20–68.4)	0.531**	< 0.001

MCV (fl)	80.1 ± 8.3 (62.4–103.3)	0.409**	< 0.001

MCH (pg/cell)	25.6 ± 4.4 (18.2–36.1)	0.430**	< 0.001

MCHC %	31.8 ± 2.7 (26.5–38.4)	0.373**	< 0.001

TLC (cells/μl)	6898 ± 2655 (2200–16,000)	0.004	0.952

ANC (cells/μl)	4696 ± 2052 (1012–12848)	-0.018	0.802

ALC (cells/μl)	1759 ± 719 (392–4200)	0.046	0.519

PLATELETS (×10^3 ^μl)	239.82 ± 101.33 (13.6–685)	0.135	0.056

RETIC %	1.17 ± 0.87 (0.5–5.5)	-0.059	0.404

S. IRON (μg/dl)	69.5 ± 52.5 (14–266)	-0.076	0.399

TIBC (μg/dl)	399.2 ± 111.3 (155–838)	0.126	0.160

S. FERRITIN (μg/dl)	421 ± 443 (5–1500)	-0.213*	0.016

SATURATION%	18.3 ± 13.9	-0.116	0.196

CD4 COUNTS (cells/μl)	202.4 ± 182 (8–1078)	1.0	

### Bone marrow abnormalities

Out of 200 patients, bone marrow aspiration was carried out in 14 (7%) individuals. Among 14 patients, two patients had non Hodgkin's lymphoma and remaining 12 patients were evaluated for pancytopenia. Six each had (42.8%, n = 6) hypocellular marrow and hypercellular marrow while 14.2% (n = 2) had normocellular marrow (14.2%). Among 12 patients with pancytopenia, bone marrow trephine biopsy revealed epithelioid cell granulomas in 6 cases (4 were positive for AFB, 2 were positive for fungal profiles, morphologically and cytochemically consistent with cryptococci), 3 cases showed hemophagocytosis and 3 cases showed no specific pathology. Staging marrow carried out in two patients with lymphoma (NHL) did not show any infiltration of lymphoma cells.

## Discussion and Conclusion

Hematological complications are a common cause of mortality in HIV infected patients. Cytopenias are most frequent during the advanced stage of disease [1]. We evaluated various hematological manifestations on 200 consecutive HIV seropositive patients who presented to the Immunodeficiency clinic PGIMER, Chandigarh, irrespective of their ART status. We also correlated the final hematological diagnosis of the patients with the CD4 count.

Anemia was the most common presentation. Among 200 individuals, 131 (65.5%) were found to be anemic out of which 4 (3%) cases were in asymptomatic group and 127 (97%) were in symptomatic group (p < 0.001). Our results on prevalence of anemia showed comparable results with other studies from India [9-11]. In a study by Mir et al on a cohort of 60 HIV infected individuals reported anemia, thrombocytopenia, leucopenia and various permutations of these in majority of individuals [12]. The highest rate of anemia occurs in patients with advanced HIV disease. In our study out of 131 patients, 92.4% (n = 121) cases were those with CD4 counts <200 cells/μL, while 7.6% (n = 10) patients were those with CD4 counts >200 cells/μL and there was statistically significant difference (p < 0.001) between both the groups. Severe anemia (defined as hemoglobin less than 7.5 gm/dl) was observed in 18.5% (n = 37) patients as compared to 7% in a study by Kasthuri et al [9].

The cumulative incidence of anemia was highest among patients who had CD4 lymphocyte count < 200 cells/μL and was lowest with CD4 lymphocyte count > 500 cells/μL, showing an inverse correlation between anemia and CD4 cell count (p < 0.001). HIV infected individuals with anemia are at increased risk for progression to AIDS and mortality, recovery from anemia has been associated with a decreased risk of deaths. We did not estimate the survival in patients with and without anemia, as this was a descriptive study, though the literature clearly indicates that it does affect the survival [13].

The hemoglobin, PCV, MCV, MCH, and MCHC showed statistically significant correlation with CD4 counts (p < 0.001) in both males and females. Iron studies were done in 126 patients out of which 65.1% (n = 82) were males and 34.9% (n = 44) were females. Iron deficiency anemia was found in 49.2% cases (n = 62; 34 males, 28 females). Anemia of chronic disease was found in 50.8% cases (n = 64; 48 males, 16 females) among which 2 males showed macrocytosis with MCV >100, a possibility of coexisting megaloblastic anemia could not be ruled out as serum vitamin B12/folic acid level was not done. Among patients with anemia 25.9% (34/131) males and 21.3% (28/131) females had iron deficiency anemia, while 36.6% (48/131) males and 12.2% (16/131) females had anemia of chronic disease. The parameters of iron profile serum iron, TIBC, and saturation were not significantly correlated but the serum ferritin level was significantly correlated to CD4 counts (p < 0.05). This result was similar to that reported in the study by Semba et al [14].

In this study we did not find any patient with hemolytic anaemia. Viral markers were also performed in anaemic patients None of the infective agents evaluated by serology, including EBV, CMV, Parvo B 19 or Hepatitis B were positive, except for 3 patients positive for Hepatitis C. These 3 patients had CD4 counts <200 cells/μL and serum ferritin more than 500 μg/dl which suggests anemia of chronic disease.

Thrombocytopenia is known to be a frequent complication of HIV infection (1, 2). The mean platelet count in our cohort was 239.8 ± 101.3 × 10^3^/μl (range 13.6–685 × 10^3^/μl). It had no significant correlation with CD4 counts confirming to the data with the previous study [10]. Prevalence of thrombocytopenia is reported to be higher among persons with AIDS, older persons, homosexuals and injecting drug users [15].

We did not detect any case of neutropenia with ANC of less than 1000 cells/mm^3^. The result differ from previous study by Suresh et al in which 22.7% cases had neutropenia [10]. We also did not find any patient with coagulation abnormalities. Bone marrow examination was carried out in 7% (n = 14) patients. Among 14 patients, 6 each had hypocellular and hypercellular marrow while 2 patients had a normocellular marrow. The bone marrow trephine biopsy showed epithelioid cell granulomas in 6 patients (4 were positive for AFB, 2 were positive for fungal profiles, morphologically and cytochemically consistent with cryptococci). Hemophagocytosis was prominently seen in 3 cases, without evidence of any co-infection. Two patients with lymphoma (NHL) showed no infiltration by lymphoma cells. HIV infection may lead to anemia in different ways; the important causes are defective iron metabolism and reutilization, nutritional deficiencies, opportunistic infections, ART, administration of chemotherapeutic agents and advanced stage of disease with its complications. Therefore, consideration should be given to monitoring anemia in all HIV-positive individuals and to offer supplements like iron preparations, vitamin B12 or folic acid, and in severe cases to provide erythropoietin treatment or blood transfusions, in case indicated.

## Competing interests

The authors declare that they have no competing interests.

## Authors' contributions

BD: Recruited the patients, collected the data, analyzed it and wrote the draft of the manuscript. AW: Conceived the study, supervised the collection of data and revised the draft. RKS: Interpreted the data collected and wrote the draft along with BD. AS: Recruited the patients, analyzed the data and revised the draft. RD: Ensured quality of the laboratory results, reported the bone marrow smears and revised the draft of the manuscript. All authors read and approved the final version of the manuscript.

## Pre-publication history

The pre-publication history for this paper can be accessed here:


